# Feasibility and safety of vertebrobasilar artery cooling infusion (VACI) following thrombectomy for acute posterior circulation ischemic stroke: A pilot randomized controlled trial

**DOI:** 10.1016/j.neurot.2026.e00894

**Published:** 2026-04-02

**Authors:** Zhe Cheng, Yuchuan Ding, Gary Rajah, Jie Gao, Fenghai Li, Linlin Ma, Jing Liu, Xiaokun Geng

**Affiliations:** a Department of Neurology and Stroke Intervention & Translational Center (SITC), Luhe Hospital, Capital Medical University, Beijing, China; b Department of Neurosurgery, Wayne State University School of Medicine, Detroit, MI, 48201, USA; c Neurosurgery and Neurovascular Surgery, Director of Comprehensive Stroke Center, Munson Medical Center, Traverse City, MI, USA; d Luhe Institute of Neuroscience, Capital Medical University, Beijing, 101100, China

**Keywords:** Large vessel occlusion, Endovascular therapy, Selective hypothermia, Neuroprotection, Stroke outcomes

## Abstract

Not all patients with acute ischemic stroke (AIS) benefit from endovascular therapy (EVT), particularly in the posterior circulation. This pilot randomized controlled trial evaluated the safety, feasibility, and preliminary efficacy of vertebrobasilar artery cooling infusion (VACI) administered after successful recanalization as a neuroprotective strategy for posterior circulation AIS. Participants were randomly assigned (1:1) to VACI or control. The VACI group received 300 mL of 4 °C saline infused into the vertebral artery at 30 mL/min after thrombectomy, whereas controls received 300 mL of 37 °C saline. All patients received standard guideline-based care. The primary endpoint was symptomatic intracranial hemorrhage (sICH). Secondary endpoints included functional outcomes, infarct volume, mortality, intracranial hemorrhage (ICH), vasospasm, coagulation abnormalities, pneumonia, and urinary tract infection. VACI following EVT appears safe and feasible in posterior circulation AIS and may offer potential neuroprotective benefit. Forty patients were enrolled and analyzed. The incidence of sICH did not differ between groups. Rates of neurological deterioration and other complications were comparable. Favorable trends were observed in the VACI group, including improved early neurological recovery (median NIHSS 6 vs. 12.5) and smaller final infarct volume (9.0 vs. 17.5 mL). Mortality was numerically lower in the VACI group at 90 days (10.0% vs. 20.0%) and at 7 days (5.0% vs. 20.0%). Although differences were not statistically significant, outcomes consistently favored VACI. VACI following EVT appears safe and feasible in posterior circulation AIS and may offer potential neuroprotective benefit.

## Introduction

Mechanical thrombectomy (MT) has substantially improved outcomes in acute ischemic stroke (AIS) caused by anterior circulation large vessel occlusion (LVO). However, the therapeutic benefit of MT in posterior circulation LVO remains less certain and continues to be actively investigated [1]. Recent randomized controlled trials, including BAOCHE and ATTENTION, have provided supportive evidence for MT in basilar artery occlusion; nevertheless, the magnitude of benefit appears more modest compared with anterior circulation stroke [2,3]. A persistent concern is the discrepancy between high rates of successful recanalization—approaching 90%—and relatively poor clinical outcomes. For example, only approximately 46% of patients achieve a modified Rankin Scale (mRS) score of 0–3 at 90 days, while mortality exceeds 30% [[2], [3], [4]]. These findings underscore a substantial therapeutic gap and highlight the need for adjunctive neuroprotective strategies to improve outcomes in posterior circulation AIS despite technically successful reperfusion.

Therapeutic hypothermia (TH) has demonstrated clinical efficacy in neonates with hypoxic–ischemic encephalopathy and in patients resuscitated after cardiac arrest, establishing hypothermia as a recognized neuroprotective intervention [[5], [6], [7]]. In AIS, TH has attracted increasing interest based on robust preclinical evidence demonstrating modulation of inflammatory cascades, metabolic demand, excitotoxicity, and apoptotic signaling pathways [8]. However, systemic or surface cooling approaches are limited by delayed intracranial temperature reduction, prolonged induction time, and frequent systemic complications [7]. These limitations may partly account for the inconsistent functional benefits observed in prior clinical trials [9]. Selective endovascular cooling offers a targeted strategy capable of rapidly inducing localized hypothermia within ischemic brain tissue while minimizing systemic adverse effects. Intra-arterial cold saline infusion following recanalization has been reported to be safe and associated with reduced infarct volume in anterior circulation AIS [[9], [10], [11]].

Despite these advances, the application of selective hypothermia in posterior circulation AIS remains insufficiently explored. Most prior clinical studies have focused predominantly on anterior circulation stroke, leaving a critical evidence gap in posterior circulation disease. To date, only limited patient numbers have been reported in posterior circulation cohorts [12], and no prospective randomized study has specifically evaluated intra-arterial regional cooling combined with MT in this population. Given the high rates of reperfusion yet persistently poor clinical outcomes in posterior circulation AIS, intra-arterial cooling may represent a rational adjunctive neuroprotective approach. Therefore, we conducted a single-center, prospective pilot randomized study to evaluate the safety, feasibility, and preliminary efficacy of vertebrobasilar artery cooling infusion (VACI) following MT in patients with posterior circulation AIS.

## Methods/Design

### Ethics approval and trial registration

The study was approved by the Ethics Committee of Beijing Luhe Hospital, Capital Medical University (Beijing, China), and was registered at the Chinese Clinical Trial Registry (www.chictr.org.cn; ChiCTR2200065806) in November 2022.

### Study design

This was a single-center, prospective, randomized controlled pilot trial designed to evaluate the safety, feasibility, and preliminary efficacy of intra-vertebrobasilar regional hypothermia following MT in patients with posterior circulation AIS treated within 8 h of symptom onset [13]. Participant safety was monitored by an independent physician not otherwise involved in the study.

### Participants and screening

Consecutive patients with acute posterior circulation large vessel occlusion (LVO) treated with MT at the stroke center of Beijing Luhe Hospital were screened for eligibility following successful recanalization.

The inclusion criteria were: 1) age between 18 and 80 years, 2) clinical diagnosis of AIS, 3) posterior circulation LVO confirmed by CTA, MRA or DSA (vertebral artery V4 segment, basilar artery, or posterior cerebral artery), 4) <8 h from symptom onset to arterial puncture, 5) baseline NIHSS (National Institutes of Health Stroke Scale) score≥8.

The exclusion criteria were:1) pre-stroke modified Rankin Scale (mRS) > 2, 2) Spontaneous vessel recanalization prior to intervention, 3) no vessel recanalization (TICI score ≤1) achieved, 4) rapid neurological recovery after vessel recanalization (NIHSS <6), 5) Extensive bilateral cerebellar infarction or bilateral extensive brainstem ischemia on imaging, 6) Severe hypertension (>185/110 mmHg), 7) Coagulation disorders, 8) blood glucose <2.7 or >22.2 mmol/L, 9) severe cardiac dysfunction, 10) pregnancy, 11) no informed consent obtained, 12) Participation in another clinical trial within 3 months.

### Enrollment, randomization and blinding

Enrollment and randomization were performed after successful recanalization. Informed consent was obtained from each participant or an appointed legal representative if the participant was lacking decision-making capacity. Eligible patients were randomly assigned in a 1:1 ratio to the VACI or control group using a computer-generated randomization sequence (total *n* = 40). The neurointerventionalist performing the procedure was not blinded due to the nature of the intervention. However, outcome assessors, data collectors, and statisticians were blinded to treatment allocation.

### Interventions

**Fig. 1:**
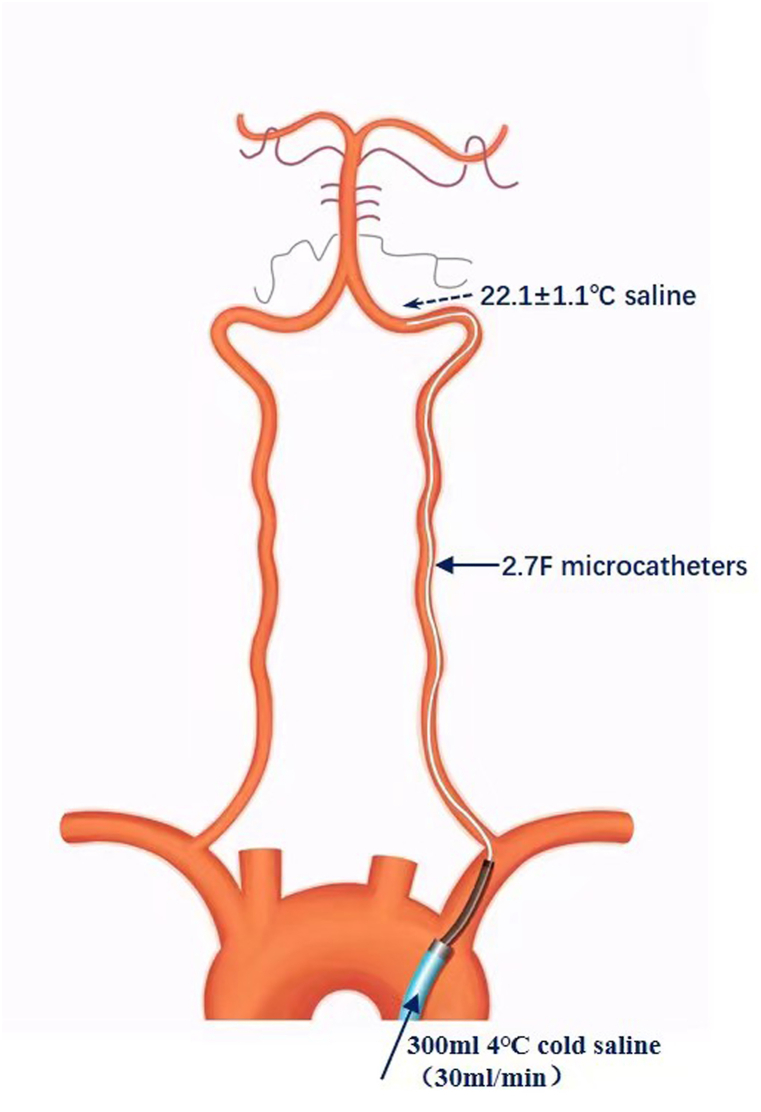
Sketch map of the VACI procedure.

All endovascular procedures were performed under local anesthesia with or without sedative agents. Following successful recanalization, VACI was administered via a 2.7F microcatheter positioned in the vertebral artery to deliver cold saline infusion as promptly as possible. Depending on the final procedural technique (e.g., stent retriever thrombectomy, self-expanding stent placement, or balloon-expandable stent placement), the microcatheter was exchanged or re-advanced into the vertebral artery when necessary. After appropriate positioning of the microcatheter, the intermediate catheter (5F/6F) was withdrawn into the subclavian artery to minimize potential interference with blood flow (Fig. 1). In the VACI group, 300 mL of 4 °C saline was infused at 30 mL/min. The control group received 300 mL of 37 °C saline at the same infusion rate. All patients received standard guideline-based medical management, including antiplatelet or anticoagulant therapy, blood pressure control, glycemic management, lipid-lowering therapy, and treatment of complications as indicated. In an *invitro* experiment, 4 °C cold saline was infused at a rate of 30 ml/min through a microcatheters (Excelsior XT-27, Stryker, 2.7F) submerged in a 37 °C water bath to simulate human blood temperature. The temperature of the saline at the catheter tip upon exit was measured at 22.1 ± 1.1 °C (*n* = 6), representing the approximate that temperature would be delivered intracranially during clinical application.

### Outcomes and assessment procedures

Demographic characteristics, vascular risk factors, comorbidities, occlusion site, procedural details, vital signs, oxygen saturation, rectal temperature, and laboratory parameters were recorded before, during, and after infusion. NIHSS scores were assessed at baseline and at 24 h, 7 days, and 90 days after stroke onset. Functional outcomes were evaluated using the modified Rankin Scale (mRS) at 24 h, 7 days, and 90 days by trained neurologists blinded to treatment allocation [14]. Non-contrast CT was performed 24 h after MT or earlier if neurological deterioration occurred. MRI with diffusion-weighted imaging was performed within 7 days to assess infarct volume, which was quantified using a Siemens Syngo.Via workstation according to a previously validated method [15]. Recanalization status was assessed using the modified Thrombolysis in Cerebral Infarction (mTICI) scale [16], by two independent neuroradiologists blinded to group allocation.

#### Primary outcomes

The primary endpoint was symptomatic intracranial hemorrhage (sICH), defined according to ECASS II criteria [17] as parenchymal hemorrhage type I or II associated with a ≥4-point increase in NIHSS score.

#### Secondary efficacy outcomes

The secondary efficacy outcomes included: 1) mRS score at 90 days, 2) favorable prognosis at 90 days (as determined by mRS scores of 0–3),3)mRS scores of 0–1 at 90 days, 4)mRS scores of 0–2 at 90 days 5) NIHSS scores at 24 h, 7 days, and 90 days, and 6) final infarct volumes.

#### Secondary safety outcomes

Included 1) mortality (mRS score 6), 2) ICH and fatal ICH, and 3) cerebral vasospasm, coagulation abnormality, pneumonia and urinary infection.

### Statistical analyses

All analyses were conducted according to the intention-to-treat principle. As a pilot study, no formal sample size calculation was performed; the sample size was chosen to evaluate feasibility and generate effect size estimates for future trials. Continuous variables were assessed for normality using the Kolmogorov–Smirnov test. Normally distributed data were compared using independent two-sided t-tests, whereas non-normally distributed data were analyzed using the Mann–Whitney *U* test. Categorical variables were compared using χ^2^ or Fisher’s exact test as appropriate. A two-sided *p* value < 0.05 was considered statistically significant. Statistical analyses were performed using Stata 17.

## Results

### Patients

**Fig. 2:**
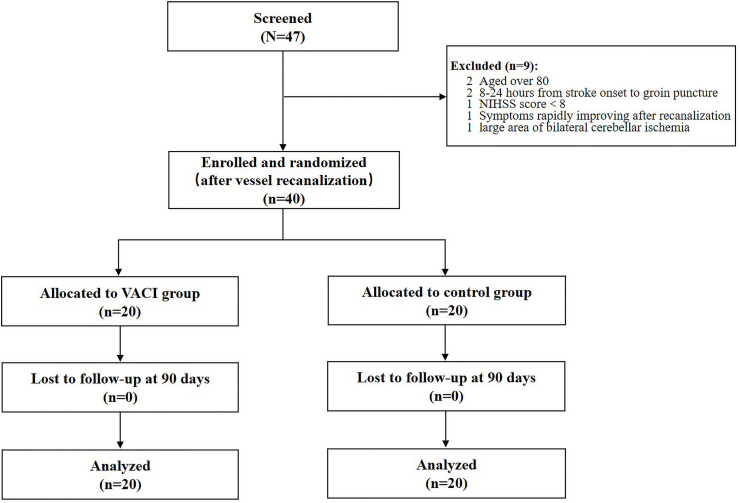
Flow and timeline of participants enrolled.

A total of 47 patients were screened and 40 patients enrolled and randomized. No participants were lost to follow-up, and all were included in the final analysis (Fig. 2). Baseline demographic and characteristics of the patients are summarized in Table 1. There were no significant differences between the hypothermia and control groups in terms of age, sex distribution, baseline NIHSS, vascular risk factors, stroke location, use of IV rt-PA, stent utilization, time from symptom onset to recanalization, time from puncture to recanalization, the number of retriever stent passes and final TICI score.

**Table 1:** Demographic and clinical characteristics of all patients.

	VACI (*n* = 20)	Control (*n* = 20)	*P Value*
Age, mean ± SD, y	61.5 ± 11.7	61.9 ± 14.1	0.923
Male, no. (%)	13 (65.0)	16 (80.0)	0.479
NIHSS score, median (IQR)	18.2 ± 6.0	19.9 ± 5.1	0.339
Risk factors, no. (%)			
*Hypertension*	15 (75.0)	14 (70.0)	0.723
*Diabetes mellitus*	7 (35.0)	8 (40.0)	0.744
*Atrial fibrillation*	4 (20.0)	6 (30.0)	0.715
*Smoking*	10 (50.0)	9 (45.0)	0.752
rt-PA, no. (%)	6 (30.0)	8 (40.0)	0.507
Basilar artery occlusion, no. (%)	16 (80.0)	16 (80.0)	1.000
Stenting/balloon dilatation, no. (%)	12 (60.0)	11 (55.0)	0.749
Time from puncture to recanalization, median (IQR), min	55 (31–89)	77 (41–98)	0.304
Time from onset to recanalization, median (IQR), min	270 (186–388)	251 (182–338)	0.718
The number of passages of retriever stent, median (IQR)	1 (1–1)	1 (1–1)	0.345
TICI≥2b, no. (%)	19 (95.0)	18 (90.0)	1.000

Abbreviations: NIHSS, the National Institute of Health Scale Score; ASPECT, Alberta Stroke Program Early Computed Tomography Score; mRS, modified Rankin score; rt-PA, intravenous recombinant tissue plasminogen activator; TICI, thrombolysis in cerebral infarction; IQR, Interquartile range.

### Safety outcomes

There was no significant difference in the incidence of sICH, the primary outcome, between hypothermia and control groups (0.0% vs. 0.0%, *p* = 1.000). Similarly, no significant difference was observed in the rates of cerebral vasospasm (5.0% v 0.0%), ICH (0.0% vs. 5.0%), fatal ICH (0.0% vs. 0.0%), pneumonia (25.0% vs. 45.0%) and urinary infection (10.0% vs. 5.0%). In addition, all measured coagulation parameters were comparable between the two groups (Table 2).

**Table 2:** Comparison of safety outcomes.

Outcome	VACI (*n* = 20)	Control (*n* = 20)	*P* Value
sICH, n (%)	0 (0.0)	0 (0.0)	1.000
Cerebral vasospasm^a^, no. (%)	0 (0.0)	0 (0.0)	1.000
ICH, n (%)	0 (0.0)	1 (5.0)	1.000
Fatal ICH, n (%)	0 (0.0)	0 (0.0)	1.000
Pneumonia, n (%)	5 (25.0)	9 (45.0)	0.185
Urinary infection, n (%)	2 (10.0)	1 (5.0)	1.000
Coagulation parameters			
*PT*	11.9 (11.5–12.8)	12.2 (11.6–16.8)	0.588
*APTT*	24.7 (23.1–27.3)	25.4 (24.0–26.6)	0.708
*TT*	15.3 (14.3–17.5)	14.4 (13.9–15.9)	0.166
*Fibrinogen*	2.8 (2.3–3.7)	3.2 (2.5–3.8)	0.296
*D-dimer*	0.7 (0.5–1.1)	0.5 (0.3–1.2)	0.322

Abbreviations: ICH, intracranial hemorrhage; sICH, symptomatic intracranial hemorrhage; PT, prothrombin time; APTT, activated partial thromboplastin time; TT, thrombin time.

a Cerebral vasospasm was assessed by angio after VACI in the study.

### Efficacy outcomes

**Fig. 3:**
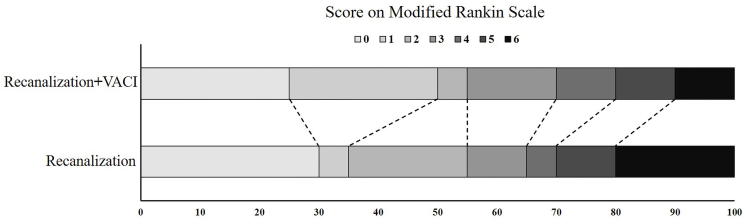
Modified Rankin Scale Score in AIS patients after vessel recanalization at 90 days. Images of Scores on the modified Rankin scale range from 0 to 6 in AIS patients after vessel recanalization at 90 days. 0 indicating no symptoms, 1 no clinically significant disability, 2 slight disability, 3 moderate disability, 4 moderately severe disability, 5 severe disability, and 6 death.

**Fig. 4:**
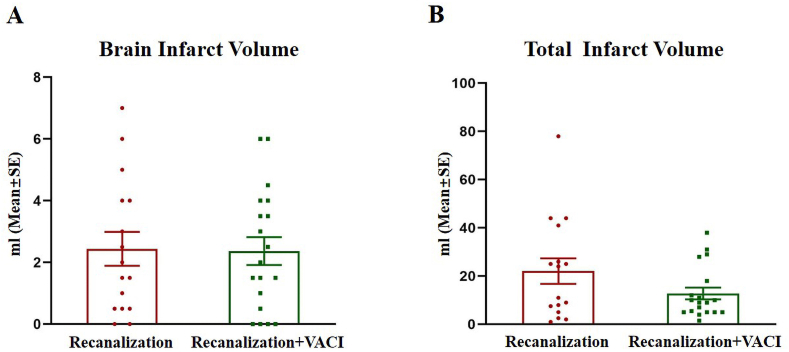
Infarct volume at 7 days after vessel recanalization. Comparison of infarct volume at 7 days after vessel recanalization between the two groups, demonstrating a decreased tendency of cerebellum infarct volume determined by MRI in the VCI group.

**Fig. 5:**
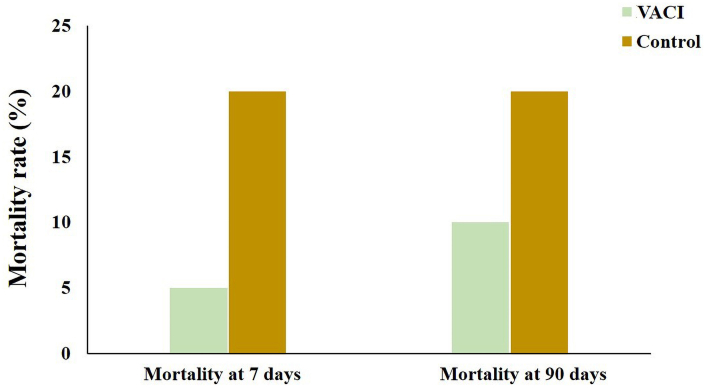
Mortality rate in AIS patients after vessel recanalization at 7 and 90 days. Comparison of mortality rate at 7 and 90 days after vessel recanalization between the two groups, demonstrating a decreased tendency of mortality rate determined in the VACI group, especially at 7 days.

Favorable trends were observed in the VACI group across multiple efficacy measures. Notably, early neurological recovery, as assessed by NIHSS at 24 h, was markedly better in the VACI group (median: 6 vs. 25), approaching statistical significance (*p* = 0.051). While the rates of functional outcome at 90 days (mRS 0–1: 50% vs. 35%; mRS 0–2: 55% vs. 55%; mRS 0–3: 75% vs. 70%) did not differ significantly between groups, these results remained consistent with the overall favorable pattern seen with VACI treatment (Table 3; Fig. 3). MRI analysis also revealed a substantial reduction in total infarct volume in the VACI group compared to controls (median: 9.0 ml vs. 17.5 ml; *p* = 0.333) (Fig. 4). Furthermore, although the difference did not reach statistical significance, the VACI group exhibited a clinically notable reduction in mortality compared to the control group at both 90 days (10.0% vs. 20.0%; *p* = 0.658) and, more prominently, at 7 days (5.0% vs. 20.0%; *p* = 0.339) (Fig. 5). These findings suggest a potential therapeutic benefit of VACI, warranting further investigation in larger studies.

**Table 3:** Comparison of efficacy outcomes.

Outcome	VACI (*n* = 20)	Control (*n* = 20)	*P* Value
mRS at 90 days, median (IQR)	1.5 (0.3–4.0)	2.0 (2.0–5.0)	0.738
mRS 0–1 at 90 days, no. (%)	10 (50%)	7 (35%)	0.523
mRS 0–2 at 90 days, no. (%)	11 (55.0)	11 (55.0)	1.000
mRS 0–3 at 90 days, no. (%)	15 (75.0)	14 (70.0)	0.723
mRS 6 at 90 days, no. (%)	2 (10.0)	4 (20.0)	0.658
mRS 6 at 7 days, no. (%)	1 (5.0)	4 (20.0)	0.339
NIHSS score at 24 h, median (IQR)	6 (3.0–12.3)	12.5 (5.0–19)	0.051
NIHSS score at 7 days, median (IQR)	4 (2–11.8)	5 (2–22)	0.569
Total infarct volume, median (IQR), ml^a^	9.0 (5.0–18.0)	17.5 (5.6–37.3)	0.333
Infarct volume of brainstem, median (IQR), ml^a^	2.0 (0.5–4)	1.8 (0.5–4)	0.909

Abbreviations: mRS, Modified Rankin Scale.

a 1 patient in Hypothermia group and 3 patients in control group did not complete the MRI scan, the mean total infarct volumes were 12.8 mL in the Hypothermia group and 22.1 ml in the control group, the mean infarct volumes of brainstem were 2.4 ml in both groups.

## Discussion

To our knowledge, this was the first study to demonstrate the safety and feasibility of intra-arterial regional cooling infusion, termed VACI, as an adjunctive therapy following endovascular recanalization in patients with AIS due to posterior large vessel occlusion. In this pilot randomized trial, VACI did not increase the incidence of sICH, nor did it elevate the risk of other safety endpoints, including overall ICH, fatal ICH, cerebral vasospasm, coagulation abnormalities, pneumonia, and urinary tract infections. These findings support the safety profile of VACI in this population. In terms of feasibility, intra-arterial delivery of 300 mL of cold saline (4 °C) via microcatheter into the vertebral artery at a controlled infusion rate was technically achievable and well tolerated, suggesting the practical viability of this endovascular cooling strategy in clinical settings.

Although this trial was not powered to assess efficacy, favorable trends were consistently observed across multiple outcome measures in the VACI group. These included improved early neurological recovery, reduced infarct volume, and numerically lower mortality at both 7 and 90 days. Functional outcome scores at 90 days also trended in favor of VACI. While these differences did not reach statistical significance, the consistency across outcomes highlights the potential therapeutic value of VACI. Taken together, these results warrant further investigation in larger, adequately powered multicenter trials to evaluate the clinical benefit of this neuroprotective strategy in posterior circulation stroke.

### Clinical advantages for regional rather than systemic cooling

Therapeutic hypothermia is widely recognized as a promising neuroprotective approach, acting on multiple pathophysiological pathways [[18], [19], [20], [21]]. Despite this potential, clinical translation of systemic hypothermia in AIS has been limited by several practical and physiological barriers. Systemic hypothermia achieved through surface cooling devices, intravenous infusion, or pharmacologic methods has demonstrated safety in patients with severe AIS but has consistently failed to produce robust functional benefits in clinical trials [[22], [23], [24]]. The primary limitations include delayed onset of cooling in brain tissue, prolonged treatment duration, and a high incidence of systemic complications such as coagulopathy, infection, shivering, and electrolyte disturbances.

In contrast, regional hypothermia via intra-arterial cooling infusion provides targeted and rapid reduction of brain temperature. Direct delivery of cold saline into the ischemic vascular territory allows selective cooling of vulnerable tissue without substantially affecting core body temperature [25,26]. Beyond temperature modulation, intra-arterial infusion may also “flush” the ischemic microcirculation, facilitating the clearance of inflammatory cells, reactive oxygen species, and toxic metabolic byproducts that accumulate during ischemia-reperfusion injury in the ischemic territory at risk^27-29^. This dual-action, targeted hypothermia combined with microvascular cleansing, may substantially reduce reperfusion-related damage while avoiding the complications of systemic cooling.

### Integration with modern reperfusion therapy

Over the past two decades, numerous neuroprotective agents demonstrating preclinical efficacy have failed in clinical translation, often due to delayed administration, insufficient brain penetration, or misalignment with evolving reperfusion strategies [[27], [28], [29], [30], [31], [32]]. With the widespread adoption of mechanical thrombectomy and high rates of successful recanalization, a therapeutic window has emerged for adjunctive interventions targeting reperfusion injury [27]. VACI represents a novel, mechanism-driven approach that complements the timing and physiology of endovascular reperfusion, which enables rapid, selective cooling of the ischemic brain territory, minimizing the delay inherent in systemic or surface cooling methods. The concept of combining regional hypothermia with MT aligns well with the current translational paradigm in stroke therapy.

### Feasibility of regional cerebral hypothermia induced by VACI

In vitro validation demonstrated that saline exiting the catheter tip reached approximately 22 °C, representing the temperature delivered intracranially under clinical infusion conditions. Direct intracerebral temperature monitoring was not performed in this pilot trial due to ethical and practical considerations. However, prior clinical and computational studies support the capacity of intra-arterial cooling to achieve meaningful regional hypothermia. For example, intracarotid infusion of 4–10 °C saline at 33 mL/min during cerebral angiography resulted in a measurable reduction in jugular bulb temperature within minutes [33]. Computational modeling has similarly demonstrated rapid decreases in regional brain temperature under comparable infusion parameters [25]. Notably, intra-arterial cooling achieves target temperature reduction substantially faster than systemic or surface cooling methods [34,35]. In the present study, VACI was well tolerated and did not increase procedural complexity or adverse events. Collectively, these findings support the technical and logistical feasibility of regional cerebral hypothermia using VACI in posterior circulation AIS, a condition in which therapeutic options remain limited and outcomes suboptimal.

### Study limitations

This pilot study has several limitations. First, it was conducted at a single center with a relatively small sample size. Although safety and feasibility were demonstrated, observed numerical differences in clinical and imaging outcomes did not reach statistical significance and should be interpreted cautiously. Second, the optimal parameters for regional cooling—including infusion volume, temperature, rate, and duration—remain to be defined. Future studies may explore prolonged cooling strategies, including maintenance infusion approaches. Third, although mechanical thrombectomy for posterior cerebral artery occlusion remains an area of ongoing debate, such patients were included in this study based on supportive evidence for endovascular therapy in selected cases and the prognostic relevance of baseline NIHSS [36,37]. Finally, direct intracranial temperature monitoring was not performed; therefore, the magnitude of regional hypothermia was inferred from *in vitro* validation and previously published physiological data.

## Conclusion and future direction

This pilot study demonstrated that VACI is safe and feasible as an adjunctive therapy following endovascular recanalization in patients with AIS due to posterior circulation LVO. While the current sample size was insufficient to establish statistical efficacy, the consistently favorable trends in neurological recovery, infarct volume reduction, and early mortality suggest a potential therapeutic benefit. To validate these preliminary findings, a larger multicenter randomized controlled trial is warranted. Such a study should be adequately powered to assess clinical efficacy, explore optimal infusion parameters—including volume, temperature, and rate—and evaluate long-term functional outcomes. If confirmed, VACI could represent a clinically viable neuroprotective strategy to improve outcomes in stroke patients with persistently high morbidity and mortality despite successful reperfusion.

## Author contributions

Zhe Cheng Carried out the study and responsibility for the integrity of the data and the accuracy of the data analysis and wrote the manuscript. Study concept and design: Yuchuan Ding, Xiaokun Geng, Zhe Cheng. Study supervision: Xiaokun Geng. Collected and analyzed the study data: Jie Gao, Fenghai Li, Jing Liu, Linlin Li. Critical revision of the manuscript for important intellectual content: Gary Rajah, Yuchuan Ding.

## Study funding

This study was supported partially by the Capital Clinical Special Projects (Z221100007422085) and National Natural Science Foundation of Beijing (7242082). The funding body had no role in the design of the study and collection, analysis, and interpretation of data and in writing the manuscript.

## Declaration of competing interest

The authors report no disclosures relevant to the manuscript.
